# Hydrophobicity Enhances the Formation of Protein-Stabilized Foams

**DOI:** 10.3390/molecules27072358

**Published:** 2022-04-06

**Authors:** Roy J. B. M. Delahaije, Peter A. Wierenga

**Affiliations:** Laboratory of Food Chemistry, Wageningen University, Bornse Weilanden 9, 6708 WG Wageningen, The Netherlands; roy.delahaije@gmail.com

**Keywords:** lysozyme, interfacial properties, molecular properties, foam properties, foam ability, foam stability

## Abstract

Screening proteins for their potential use in foam applications is very laborious and time consuming. It would be beneficial if the foam properties could be predicted based on their molecular properties, but this is currently not possible. For protein-stabilized emulsions, a model was recently introduced to predict the emulsion properties from the protein molecular properties. Since the fundamental mechanisms for foam and emulsion formation are very similar, it is of interest to determine whether the link to molecular properties defined in that model is also applicable to foams. This study aims to link the exposed hydrophobicity with the foam ability and foam stability, using lysozyme variants with altered hydrophobicity, obtained from controlled heat treatment (77 °C for 0–120 min). To establish this link, the molecular characteristics, interfacial properties, and foam ability and stability (at different concentrations) were analysed. The increasing hydrophobicity resulted in an increased adsorption rate constant, and for concentrations in the protein-poor regime, the increasing hydrophobicity enhanced foam ability (i.e., interfacial area created). At higher relative exposed hydrophobicity (i.e., ~2–5 times higher than native lysozyme), the adsorption rate constant and foam ability became independent of hydrophobicity. The foam stability (i.e., foam collapse) was affected by the initial foam structure. In the protein-rich regime—with nearly identical foam structure—the hydrophobicity did not affect the foam stability. The link between exposed hydrophobicity and foam ability confirms the similarity between protein-stabilized foams and emulsions, and thereby indicates that the model proposed for emulsions can be used to predict foam properties in the future.

## 1. Introduction

It is well known that different proteins show different foam properties. Understanding which molecular parameters are causing these differences is essential to eventually predict the foam properties. At this moment, the foam properties of a given protein sample cannot be estimated based on the molecular properties. The same could be said for protein-stabilized emulsions, but recently a model was introduced to describe and predict the emulsion properties based on the protein molecular properties, e.g., size, charge and exposed hydrophobicity, and protein concentration [1]. The model proposed for emulsions is expected to be applicable to foams as well, since the fundamental mechanism for foam and emulsion formation is very similar (i.e., proteins need to adsorb and saturate the interface to arrest bubble destabilisation). Recent studies indeed showed that an increase in protein concentration had a similar effect on the formation of β-lactoglobulin and ovalbumin emulsions and foams [2,3], as shown by the decrease in droplet and bubble size, for example. While these studies showed qualitative similarities, the relations between the foam and the molecular properties were not discussed. This work aims to link the exposed hydrophobicity—an important molecular characteristic (altered by controlled heat treatment)—with the foam properties of the hen egg white protein lysozyme.

### 1.1. Foam Formation

In the recently developed model, the efficiency of a protein’s ability to form a foam is assumed to mainly depend on the time required to adsorb and saturate the air–water interface [4], thereby arresting bubble coalescence in the timescale of foam formation. In other words, foam formation is mainly determined by the flux of protein molecules to the interface. This flux (dΓ/dt) is mainly affected by the number of protein molecules available to adsorb, i.e., protein concentration (C_p_), and the protein’s affinity to adsorb, which is quantified by the adsorption rate constant (k_adsorb_; Equation (1)) [5]. It should be noted that, as the interface becomes more and more saturated, the chance for a new protein to arrive at an empty spot (P_adsorb_) decreases.

(1) $$\frac{d\Gamma }{\mathrm{dt}}={\mathrm{DC}}_{p}P_{\mathrm{adsorb}}k_{\mathrm{adsorb}}$$

where Γ is the adsorbed amount [mg m^−2^], D is the diffusion coefficient in the aqueous phase [m^2^ s^−1^] and P_adsorb_ is the probability for adsorption [–] [6], which is, for all proteins, dependent on surface coverage in a similar way (i.e., identical for all proteins).

#### 1.1.1. Effect of Protein Concentration

Protein concentration significantly affects the foam formation, as was shown for β-lactoglobulin and ovalbumin [2,3]. In fact, two distinct concentration regimes were observed, namely the protein-poor and protein-rich regimes. The protein-poor regime is characterized by the fact that insufficient proteins adsorb to the interface within the timespan of foam formation. As a result, the interface is not saturated, leading to coalescence during formation. In the protein-poor regime, an increase in the protein concentration (i.e., number of available protein molecules) results in an increase in the foam volume and/or a decrease in the bubble radius. With increasing C_p_, three sub-regimes were identified in the protein-poor regime; (I) no foam, (II) increasing foam volume (reached after given time of foaming) until maximum/set foam volume was reached, and (III) decreasing bubble radius (at maximum foam volume) until the/a minimum bubble radius (r_3,2,_ min) was reached. In the protein-rich regime (regime IV), sufficient protein molecules are available to saturate the interface of all bubbles within the timescale of foam formation. Consequently, coalescence during formation was arrested, i.e., the maximum foam volume and minimum bubble radius were reached. The foam volume and bubble radius depend on system parameters, such as the pore size of the frit, the gas flow rate, and the sparging time. To describe the effect of parameters such as C_p_ on the foam formation, two critical concentrations were defined. The (critical) concentration above which the foam volume becomes concentration-independent is denoted as C_cr, FA_ (i.e., transition from regime II to III). The other (critical) concentration demarcates the transition to the protein-rich regime where the bubble radius becomes concentration-independent as well, denoted as C_cr, r3,2_ (i.e., transition from regime III to IV).

#### 1.1.2. Effect of Adsorption Rate Constant (k_adsorb_)

The affinity of a protein to adsorb to an interface (i.e., k_adsorb_) depends on the protein charge and hydrophobicity, as these parameters influence the barrier for adsorption [5,7]. Whereas an increase in charge leads to a higher adsorption barrier, an increased hydrophobicity lowers the adsorption barrier. A lower adsorption barrier (i.e., increased hydrophobicity or lower charge) results in an increased k_adsorb_ (Equation (2)) [5].

(2) $$k_{\mathrm{adsorb}}=e^{\frac{-\Delta E_{\mathrm{barrier}}}{k_{B}T}}$$

where ΔE_barrier_ is the energy barrier for adsorption [J], k_B_ is the Boltzmann constant [1.38∙10^−23^ J K^−1^], and T is temperature [K].

An increased protein exposed hydrophobicity was experimentally confirmed to result in faster adsorption (i.e., faster increase in surface pressure and adsorbed amount versus time) [7,8,9]. The expected result of exposed hydrophobicity and the consequent faster adsorption on foam formation has, however, not yet been clearly established. One possible reason is that most studies altered the hydrophobicity of the protein by heat treatment. These studies often concluded that heating of globular proteins enhanced the foam ability [10,11,12,13,14,15,16,17]. This was generally ascribed to protein unfolding and the consequent increased hydrophobicity [10,11,13,17]. A heat treatment may however also have induced well known “side” reactions, such as aggregation [18,19], which could have interfered. In other studies, the aggregation of proteins was specifically included in the analyses. Aggregates of β-lactoglobulin were for example found to either enhance or reduce the formation of β-lactoglobulin foams [17,20]. Other studies focused on natural variation in the hydrophobicity of proteins. This approach circumvents the drawbacks of heat treatment. However, differences in other protein molecular properties affecting the foam ability of protein, most importantly protein charge, were not taken into account [21,22]. As a result, there is still a need to study the contribution of exposed hydrophobicity on the foam properties.

### 1.2. Foam Stability

Besides foam formation, foam stability is an important characteristic of the foam. Typically, the foam stability is described to depend on coalescence, due to rupture of the liquid films separating the bubbles, or Ostwald ripening [23,24,25]. In the Ostwald ripening process, gas from smaller bubbles diffuse to the larger bubbles. However, in many “wet” protein foams (i.e., foams with a liquid as continuous phase) with relatively short life times, the coalescence will be the more relevant and distinguishing feature [23,26]. Based on the classical theories, coalescence depends mostly on the drainage of liquid from the thin liquid films [27,28,29]. At least, for this moment, we will consider that all proteins are dissolved as molecules, so we will not consider any effects of aggregates that may work as destabilising (anti-foam) or additional stabilising agents. The adsorbed protein layer will influence drainage due to the interactions between adsorbed layers [30,31]. At a pH away from the isoelectric point, where all proteins are similarly charged, the two interfaces in a thin liquid film will have a net repulsive interaction. This repulsive energy results in a disjoining pressure that will increase as the thin liquid film becomes thinner (as a result of drainage). In principle, drainage will stop when the capillary pressure (from the plateau borders) is in equilibrium with the disjoining pressure [30]. Rupture of the thin liquid film is supposed to occur when the thin liquid film has drained to a certain critical thickness. However, it should be noted that while this description is often described and repeated in the literature, there is still not sufficient evidence and understanding of these phenomena to quantitatively describe or predict foam stability. When considering the effect of hydrophobicity on foam stability, it may be expected that the disjoining pressure would decrease. This would lead to lower film thickness and thereby would likely be expected to decrease foam stability. However, on the other hand, there is also an influence of the mobility of the interfacial layer. In the extreme case of particle-stabilised interfaces, the interfaces are sterically “jammed”. As a result, bubbles in such a foam do not show fast coalescence, and such foams have been reported to have lifetimes in the order of days and weeks [32,33,34]. Additionally, hydrophobin, more specifically class II hydrophobin HFBII, was described to make such solidified interfaces and foams with equally long lifetimes [35]. For low-molecular-weight surfactants, such as sodium dodecyl sulphate, it has often been suggested that the high mobility of the adsorbed surfactants, reflected by the very low surface dilatational elastic modulus, would enable easy coalescence [29,36]. For proteins, one could then, based on the classical theories, expect that increased hydrophobicity would lead to higher surface dilatational elastic moduli, which could perhaps increase the stability against coalescence. It should however be noted that only very limited data, if any, is available on the effect of exposed hydrophobicity of proteins on foam stability.

This study aims to link the exposed hydrophobicity with the foam ability and stability of the hen egg white protein lysozyme. Different lysozyme variants with increasing levels of hydrophobicity were obtained from controlled heat treatment of lysozyme at 77 °C for 0–120 min. To establish this link, the molecular characteristics, interfacial properties, and foam ability and stability (at different concentrations) were analysed.

## 2. Results and Discussion

### 2.1. Structural Characterization

**Figure 1:**
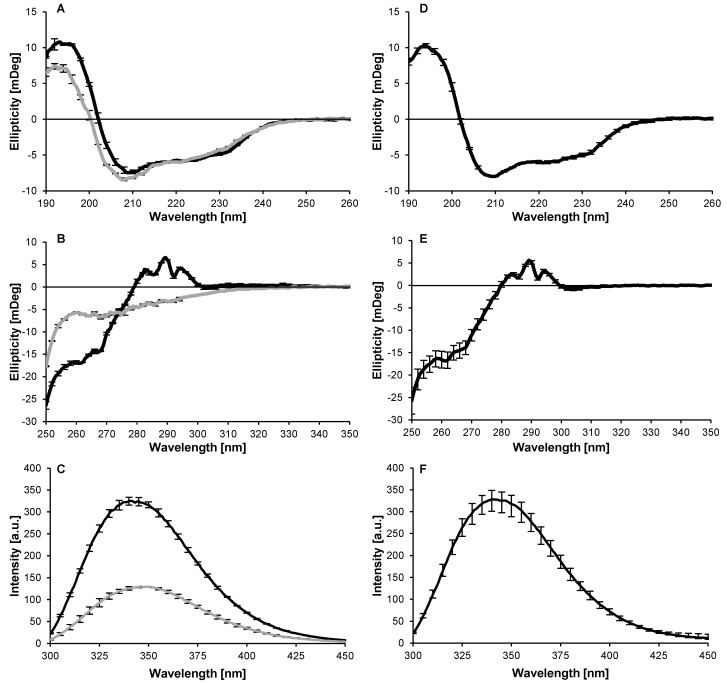
Far-UV (**A**,**D**) and near-UV (**B**,**E**) CD and intrinsic fluorescence (**C**,**F**) spectra of lysozyme at pH 5.7 and 20 °C or 77 °C → 20 °C (black) and 77 °C or 77 °C after 30 min (grey; **A**–**C**), and spectra averaged for all (un-)heated lysozyme variants (**D**–**F**; heated for 0–120 min at 77 °C) at pH 5.7 and 20 °C. The data in the panels are average values with the error bars indicating the standard deviation.

The denaturation temperature of lysozyme was found to be 74.9 °C (data not shown). This is in close agreement with denaturation temperatures reported in previous studies [37,38,39,40,41]. Concordantly, the unfolding of the secondary and tertiary structure of lysozyme occurred at 77 °C (Figure 1A–C).

**Figure 2:**
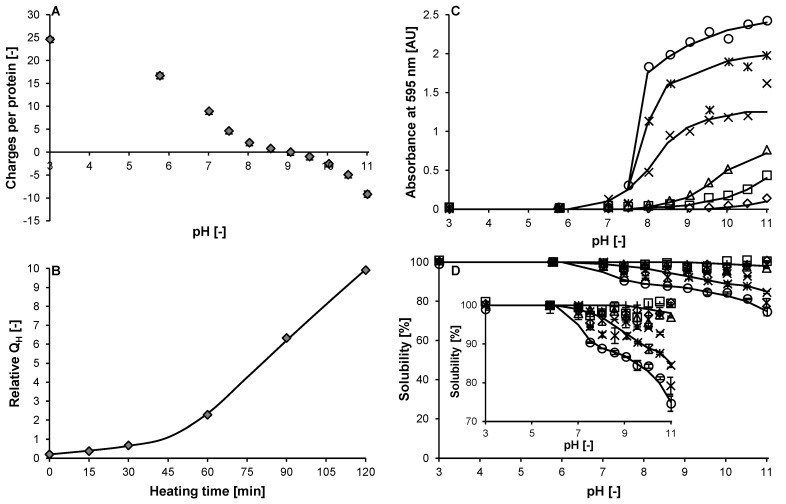
Effect of heating at 77 °C on the number of charges per lysozyme molecule as a function of pH (**A**), the relative exposed hydrophobicity (Q_H_) as function of heating time (**B**), and the turbidity (**C**) and solubility (**D**) as function of pH for (un-)heated lysozyme; 0–120 min (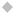), 0 (◇), 15 (☐), 30 (△), 60 (×), 90 (🞶), and 120 min (○—10 mM sodium phosphate buffer pH 5.7). The error bars in panel A indicate the standard deviation. The solid lines in panel B–D are guides to the eye. The insert in panel D shows the data with rescaled *y*-axis.

Upon cooling, lysozyme refolded into a native(-like) state, i.e., with secondary and tertiary structure similar to that of the native state (Figure 1A–C). This is in line with a previous study, which showed a high degree of refolding for lysozyme under similar conditions (i.e., pH << pI and low ionic strength) [41]. Even after prolonged heating (i.e., up to 120 min), lysozyme refolded into a native(-like) structure after cooling (Figure 1D–F). In addition, no aggregation or changes in molecular weight (Figure S1) and no changes in charge density were observed (Figure 2A).

Only the relative exposed hydrophobicity significantly increased upon heating, from 0.2 for unheated lysozyme to 9.9 after a heat treatment of 120 min (i.e., Lys120; Figure 2B). This difference indicates that lysozyme did not refold into its native state, but into a conformation which closely resembles the native state. With an increase in pH (from 5.7 to 11.0), the heated lysozyme variants—with a higher exposed hydrophobicity—showed a higher turbidity and lower solubility, even under conditions with sufficient electrostatic repulsion (i.e., pH well below the isoelectric point (pI) ≈ 11.0 [42,43,44]; Figure 2C,D). From the detailed characterisation of the molecular properties, no differences between the native and heated lysozyme were observed. It seems that lysozyme, after the heat treatment, refolded to a structure that was very similar to the native structure. A similar behaviour was previously reported for β-lactoglobulin [45]. It is assumed that lysozyme adopts a non-native but native-like structure after cooling down, where minor differences in the ordering of the structural elements—not reflected in the analysis—cause a change in exposed hydrophobicity.

### 2.2. Adsorption Kinetics

**Figure 3:**
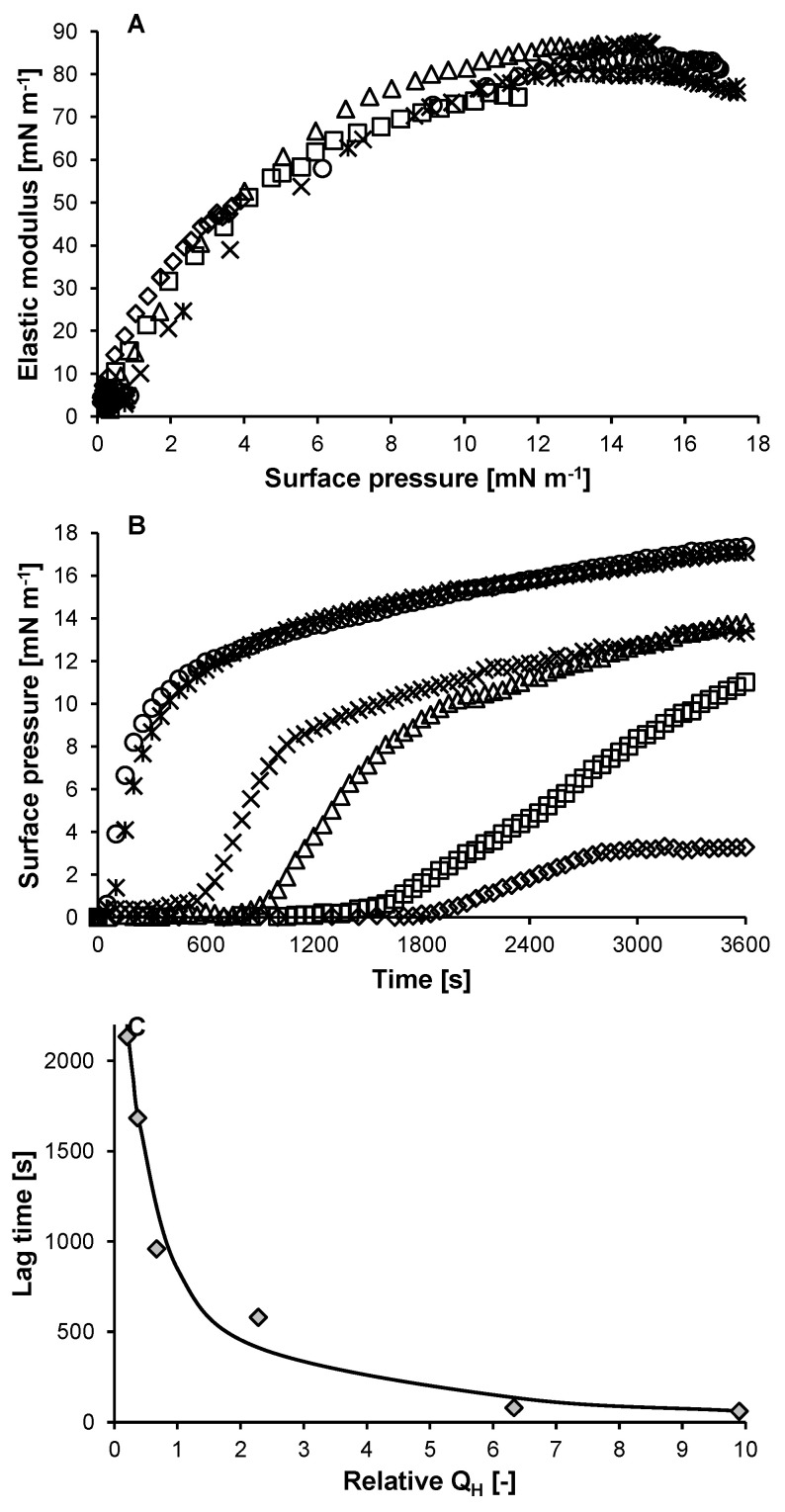
Elastic modulus as a function of surface pressure (**A**), surface pressure as a function of time (**B**) for lysozyme heated at 77 °C for 0 (◇), 15 (☐), 30 (△), 60 (×), 90 (🞶), and 120 min (○), and lag time as function of the relative exposed hydrophobicity for lysozyme heated at 77 °C for 0–120 min (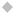) (**C**) (0.1 g L^−1^, 10 mM sodium phosphate buffer pH 5.7, 20 °C). The solid line in C is a guide to the eye.

The elastic modulus (E_d_) as a function of surface pressure (Π) overlaps for all (un-)heated variants (Figure 3A). Therefore, the change in surface pressure as a function of time (dΠ/dt) can be interpreted as the initial adsorption rate (dΓ/dt).

For most samples, the surface pressure only increased after a lag time (i.e., time required to reach a Π of 1 mN m^−1^; Figure 3B). Therefore, the lag time of adsorption was taken as an indication of the adsorption rate constant. The lag time shows a strong relation to exposed hydrophobicity (Figure 3C). This relation is in line with previous observations [7,8,9]. When the exposed hydrophobicity of the protein is relatively high (i.e., >±5; Lys90 and Lys120), a further increase in hydrophobicity only leads to a minimal further increase in adsorption rate constant (Figure 3C). This is because, at some point, the energy barrier for adsorption (ΔE_barrier_) becomes close to or even lower than thermal energy, so exp(−ΔE_barrier_/k_B_T) (Equation (2)) becomes close to 1.

### 2.3. Foam Ability

#### 2.3.1. Effect of Protein Concentration

**Figure 4:**
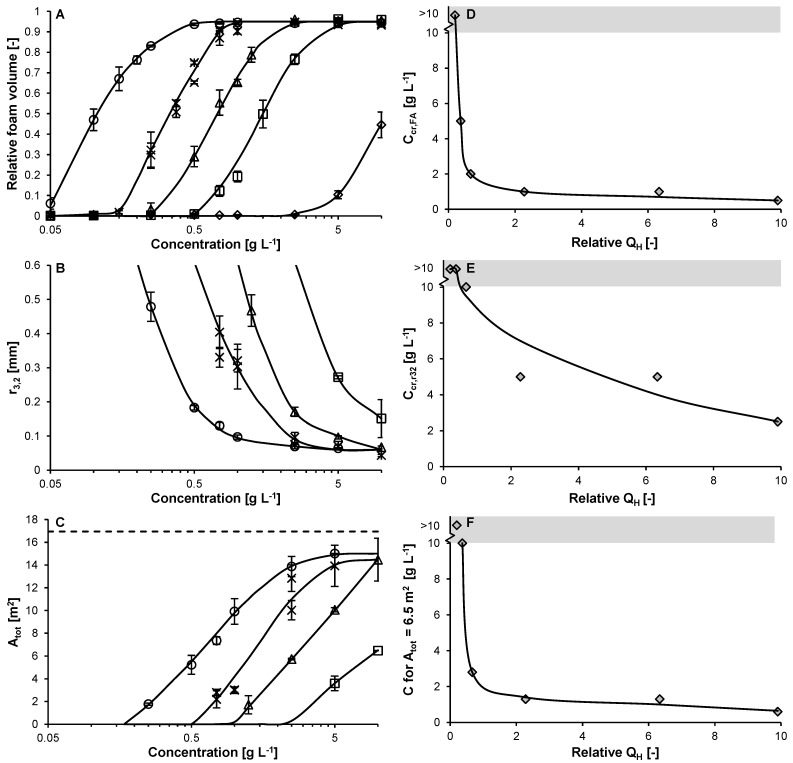
Foam ability based on the relative foam volume after sparging for 60 s relative to the foam volume of a 10% (*w*/*v*) SDS solution (**A**), bubble radius (**B**), and total interfacial area created (**C**) for (un-)heated lysozyme; 0 (◇), 15 (☐), 30 (△), 60 (×), 90 (🞶), and 120 min (○). Critical concentration for foam ability (C_cr,FA_; **D**), bubble size (C_cr,r3,2_; **E**), and concentration to reach a total interfacial area of 6.5 m^2^ for all (un-)heated variants (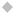; 10 mM sodium phosphate buffer pH 5.7, 25 °C; **F**). The solid lines are guides to the eye. The error bars in panel A–C indicate the standard deviation. The dashed line in panel C represents the theoretical maximum area created based on 10% (*w*/*v*) SDS solution, and the grey area in panels D–F represent critical concentrations exceeding the experimental protein concentration of 10 g L^−1^.

For all (un-)heated variants, four concentration regimes could be distinguished. Since native lysozyme showed poor foam ability at all concentrations, Lys90 is used to illustrate the effect of concentration on foam ability. For lys90, no foam was formed for concentrations ≤ 0.15 g L^−1^ (regime I; Figure 4A).

In regime II, the volume of Lys90-stabilized foam increased with increasing protein concentration and reached the maximum foam volume at 1 g L^−1^ (i.e., C_cr_,_FA_; Table 1).

At even higher concentrations (regime III), the bubble size decreased to r_3,2, min_, which was reached at a concentration of 5 g L^−1^ (i.e., C_cr,r3,2_). At concentrations ≥ 5 g L^−1^, i.e., in the protein-rich regime, the foam volume and bubble size are independent of the protein concentration (Figure 4A,B). These four concentration regimes were also reported for β-lactoglobulin and ovalbumin foams [2,3], with C_cr,3,2_ of 1 g L^−1^ and 25 g L^−1^, respectively (at pH 7). The fact that these globular proteins show similar trends in foam formation versus concentration indicates a (more) generic characteristic in the formation of protein-stabilized foams. Moreover, it clearly shows that conclusions drawn on foam properties of a specific protein strongly depend on the protein concentration used for comparing different conditions and/or proteins.

From the foam volume and r_3,2_, the total created surface area was calculated (Equation (6)). The surface area increases logarithmically with concentration until it levels off close to the theoretical maximum surface area (Figure 4C). This correlation was in line with previous observations for ovalbumin [3].

#### 2.3.2. Effect of Exposed Hydrophobicity

The critical concentration for foam ability (C_cr,FA_) showed a clear dependence on the relative exposed hydrophobicity (Figure 4D). The C_cr,FA_ decreased steeply with increasing hydrophobicity until it levelled off at a C_cr,FA_ ~1 g L^−1^ for a relative exposed hydrophobicity of ≥2 (i.e., Lys60–Lys120). Similar to C_cr,FA_, the critical concentration for bubble size (C_cr,r3,2_) also decreased with increasing hydrophobicity (Figure 4E). However, the relation between C_cr,r3,2_ and hydrophobicity was less obvious. When combining the effect of hydrophobicity on foam volume and bubble radius by expressing the concentration required to create a certain total interfacial area (in this case 6.5 m^2^) as function of exposed hydrophobicity (Figure 4F), a very similar trend was obtained as for C_cr,FA_ (Figure 4D). More specifically, the concentration decreased steeply with increasing hydrophobicity until it levelled off for a hydrophobicity ≥ 2 (i.e., Lys60–Lys120). This decrease was attributed to an increased adsorption rate constant (with increasing hydrophobicity). In this perspective, the declining effect of hydrophobicity at higher relative exposed hydrophobicity (resulting in the levelling off) is in line with prior observations for the adsorption rate constant (Figure 3C). These observations clearly show that an increase in hydrophobicity—through its effect on the adsorption barrier and thereby also on the adsorption rate constant—strongly enhances foam formation, until the adsorption barrier hardly changes at higher relative exposed hydrophobicity.

### 2.4. Foam Stability

**Figure 5:**
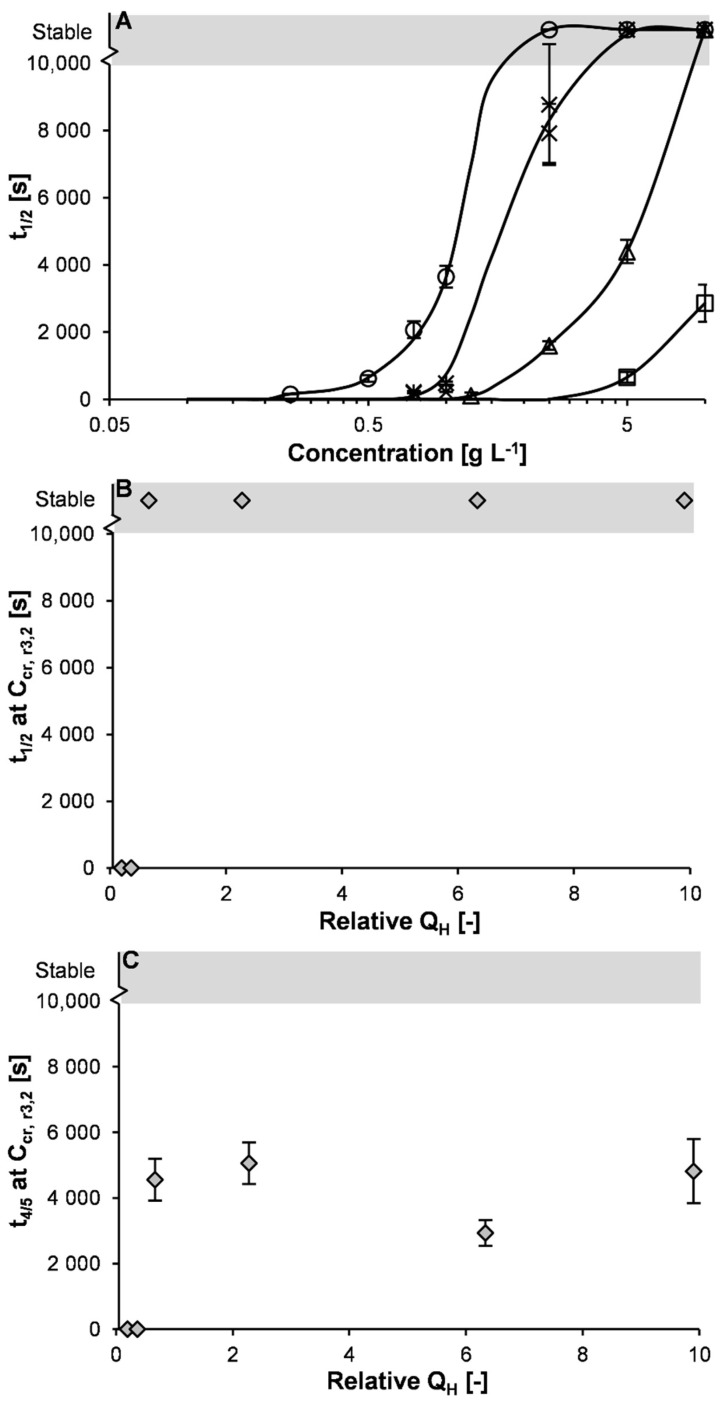
Foam half-life time (t_1/2_) as a function of concentration for (un-)heated lysozyme (**A**); 0 (◇), 15 (☐), 30 (△), 60 (×), 90 (🞶), and 120 min (○), and t_1/2_ (**B**) and t_4/5_ (**C**) at C_cr, r3,2_ as function of relative exposed hydrophobicity for all (un-)heated variants (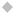; 10 mM sodium phosphate buffer pH 5.7, 25 °C). The error bars in panel A indicate the standard deviation and the solid line is a guide to the eye. The grey area indicates a foam half-life time exceeding the experimental time of 10,000 s.

The foam stability, expressed by the foam half-life time, t_1/2_, increased with concentration (e.g., for Lys120, from 160 s at 0.25 g L^−1^ to >10,000 s at ≥2.5 g L^−1^; Figure 5A). A concentration dependence of foam stability has been previously reported for ovalbumin foams, 900 s at 0.5 g L^−1^ to 6780 s at 10 g L^−1^ [3].

In addition, the foam stability at a given concentration increased significantly with increasing hydrophobicity (e.g., at 5 g L^−1^ from 655 s for Lys15 to 4400 s for Lys30 to >10,000 s for Lys60–Lys120). However, foams stabilized by variants with a higher hydrophobicity also had a smaller initial bubble size, indicating that the interfaces were closer to saturation. In the protein-rich regime (i.e., at C ≥ C_cr,r3,2_), where there are no differences in initial foam volume and bubble size, no difference in foam stability was observed, as indicated by a similar t_1/2_ as well as t_4/5_, i.e., t_1/2_ was >10,000 s and t_4/5_ was ~4810 s (Figure 5B,C). This shows that differences in foam stability in the protein-poor regime were not directly related to hydrophobicity but were caused by differences in foam volume and initial bubble size. The similar foam stability in the protein-rich regime shows that under identical (or at least very similar) conditions differences in hydrophobicity do not dominate foam stability. The differences between the effect of hydrophobicity and foam stability in the protein-poor and protein-rich regime show the importance of using identical systems (or at least reduce all differences as much as possible) when establishing relations.

Data from thin liquid films stabilized by very similar (un-)heated lysozyme variants, i.e., lysozyme heated in 10 mM phosphate buffer pH 5.7 at 5 g L^−1^ and 75 °C for 0, 30, 60, 90, and 120 min, were used for comparison (Figure S2A). For all variants, the rupture times of the thin films increased with increasing concentration (for Lys60, from ~20 s at 0.17 g L^−1^ to ~200 s at 0.5 g L^−1^). Moreover, with increasing heating time the rupture times increased (at 0.25 g L^−1^ from ~20 s for Lys0 to ~1700 s for Lys90 and Lys120). These data show that, similar to foam stability at a given concentration, the stability of a single thin film (at a given concentration) is also affected by increased concentration and hydrophobicity. Interestingly, after exchange of the liquid between the interfaces in the thin film by buffer solution, as described previously [46], the rupture time for all variants decreased to the same value (i.e., in the range 20–100 s; Figure S2B). This indicates that the stability of the thin films, and possibly also the foam, is significantly affected by the non-adsorbed proteins in the foam lamellae. Based on the thin film experiments, an increase in foam stability with increased hydrophobicity was perhaps expected, even in the protein-rich regime. This discrepancy between foam and thin film stability could be due to differences in forces at play in the capillary cell and in a foam, or differences in experimental conditions, e.g., adsorption times.

## 3. Materials and Methods

### 3.1. Materials

Lysozyme from chicken egg white (L6876, Lot nº SLBL7146V; protein content of 93.9% determined based on nitrogen content by Dumas (N × 5.29) [47], of which ≥95% is lysozyme as confirmed by RP-UPLC) and β-lactoglobulin from bovine milk (L0130, Lot nº SLBK5901V; protein content of 92.0 % determined based on nitrogen content by Dumas (N × 6.33) [48]) were purchased from Sigma-Aldrich (St. Louis, MO, USA). All other chemicals were of analytical grade and purchased from either Sigma-Aldrich or Merck (Darmstadt, Germany).

### 3.2. Denaturation Temperature

The denaturation temperature of lysozyme was determined using differential scanning calorimetry (DSC) on a VP-DSC Micro-Calorimeter (MicroCal Inc., Northampton, MA, USA), as described previously [49]. Lysozyme was dissolved in 10 mM sodium phosphate buffer pH 5.7 in a concentration of 5 g L^−1^. Subsequently, thermograms were recorded from 20 to 100 °C at a heating rate of 1 °C min^−1^.

### 3.3. Heat Treatment

Lysozyme was dissolved in 10 mM sodium phosphate buffer pH 5.7 in a concentration of 30 g L^−1^. The pH of the lysozyme solution was adjusted to 5.7 with 0.5 M NaOH. Then, the solution was transferred into Kimax tubes and heated at 77 °C for 0, 30, 60, 90, or 120 min (further referred to as Lys0–Lys120 according to the heating time). Subsequently, the solutions were cooled down under running tap water and stored at −20 °C.

### 3.4. Sample Preparation

For analysis, the (un-)heated lysozyme solutions were diluted with 10 mM sodium phosphate buffer pH 5.7 to the concentration stated in the respective analysis section (unless stated otherwise).

### 3.5. Secondary and Tertiary Structure

Far- and near-UV circular dichroism (CD): The (changes in the) secondary and tertiary structure of (un-)heated lysozyme were determined by far- and near-UV circular dichroism, respectively. The (un-)heated lysozyme solutions with concentrations of 0.1 or 1 g L^−1^ were used for far- and near-UV CD, respectively. Measurements were performed using a Jasco J-715 spectropolarimeter (Jasco Corp., Japan) with a sensitivity of 100 mDeg and a bandwidth of 2 nm. Far- and near-UV CD measurements were performed in quartz cuvettes with optical path lengths of 1 and 10 mm, respectively. The spectra of lysozyme and the heated variants (i.e., Lys0-Lys120) were recorded at 20 °C from 190 to 250 nm (i.e., far-UV CD) and from 250 to 350 nm (i.e., near-UV CD) and averaged 10 runs. After subtracting the spectra of the protein-free sample from the far-UV spectra of the sample, the relative content of secondary structure elements was estimated using a nonlinear least squares fitting procedure, as described by de Jongh et al. [50]. In addition, spectra were recorded for Lys0 before, during, and after heating in the spectropolarimeter. First, Lys0 was measured at 20 °C, followed by a measurement at 77 °C after equilibration for 5 min. The solution was kept at 77 °C for 30 min and measured once more. Finally, the solution was, after equilibration for 5 min, measured at 20 °C.

Intrinsic Fluorescence: As indication for changes in the tertiary structure, the exposure of tryptophan and tyrosine residues was determined using intrinsic fluorescence. The (un-)heated lysozyme solutions (i.e., Lys0-Lys120, at 1 g L^−1^) were excited at 280 nm and emission spectra were collected from 300–450 nm using a Varian Cary Eclipse fluorescence spectrophotometer (Agilent Technologies, Santa Clara, CA, USA). The emission and excitation slits were set to 5 nm, the scan speed was 600 nm min^−1^, the detector voltage was 600 V and the temperature was 25 °C. In addition, spectra were recorded for Lys0 before, during, and after heating in the fluorimeter. First, Lys0 was measured at 25 °C, followed by a measurement at 77 °C after equilibration for 5 min. The solution was kept at 77 °C for 30 min and measured once more. Finally, the solution was, after equilibration for 5 min, measured at 25 °C.

### 3.6. Size-Exclusion Chromatography

The molecular weight distribution was determined by size-exclusion chromatography (SEC) on an Äkta Micro equipped with a Superdex 75 PC 3.2/30 column (GE Healthcare, Uppsala, Sweden). The (un-)heated lysozyme solutions were diluted with 10 mM sodium phosphate buffer pH 5.7 + 100 mM NaCl to a concentration of 1 g L^−1^. These solutions (50 µL) were injected and eluted with 10 mM sodium phosphate buffer pH 5.7 containing 100 mM NaCl at a flow rate of 0.08 mL min^−1^. The elution was monitored using UV absorbance at 280 nm. The column was calibrated with globular proteins with a mass range of 13.7–67 kDa.

### 3.7. Mass Spectrometry

The molecular weight of the (un-)heated lysozyme was determined using an H class ACQUITY UHPLC System with an online SYNAPT G2-Si high-definition mass spectrometer using the gradient and MS settings, as described previously [51]. The (un-)heated lysozyme solutions were diluted in eluent A (i.e., 1 % (*v*/*v*) acetonitrile (ACN) containing 0.1 % (*v*/*v*) trifluoracetic acid (TFA) in milliQ water) to a final concentration of 1 g L^−1^, followed by centrifugation (16100× *g*, 10 min). The supernatants (2 μL) were injected.

### 3.8. Surface Charge

The pH of the (un-)heated lysozyme solutions (10 g L^−1^) was adjusted with 0.5 M HCl to pH 3.0, and subsequently with 0.5 M NaOH to pH 7.0, 7.5, 8.0, 8.5, 9.0, 9.5, 10.0, 10.5, and 11.0. The number of charges per lysozyme molecule was calculated from the protein concentration and the added volume NaOH using Equation (3).

(3) $$\sigma =\frac{c_{\mathrm{NaOH}}\cdot V_{\mathrm{NaOH}}-\left(10^{-\mathrm{pH},\;\mathrm{initial}}\cdot V_{\mathrm{total},\;\mathrm{initial}}-10^{-\mathrm{pH},\;\mathrm{final}}\cdot V_{\mathrm{total},\mathrm{final}}\right)}{\frac{c_{\mathrm{protein},\;\mathrm{initial}}}{M_{w}}\cdot V_{\mathrm{total},\mathrm{initial}}}$$

where σ is the charge per lysozyme molecule [mol mol^−1^], c_x_ is the concentration of x [mol L^−1^], V_x_ is the volume of x [L], and pH_x_ is the pH at moment x [-]. Subscripts initial and final refers to the situation prior to or after pH change, respectively. The protein concentration [mol L^−1^] was calculated based on the M_w_ of lysozyme (i.e., 14,313 g/mol; uniprot.org entry code: P00698).

### 3.9. Exposed Hydrophobicity

The increase in fluorescence intensity upon binding of 8-anilino-1-napthalenesulfonic acid (ANSA) to the accessible hydrophobic regions of the protein was used as a measure of the protein surface hydrophobicity [52]. The (un-)heated lysozyme solutions were diluted to a concentration of 1 g L^−1^. β-Lactoglobulin was dissolved in 10 mM sodium phosphate buffer pH 5.7 in a concentration of 1 g L^−1^. The measurements were performed on a Varian Cary Eclipse fluorescence spectrophotometer (Agilent Technologies, Santa Clara, CA, USA) as described elsewhere [7]. The excitation wavelength was set to 385 nm and the emission spectrum was measured from 400 to 650 nm at 25 °C. The fluorescence spectrum with the highest area was corrected with the area of the buffer. Subsequently, the relative exposed hydrophobicity (Q_H_) was expressed as the area of the sample relative to the area of β-lactoglobulin.

### 3.10. Turbidity and Solubility

The pH of the (un-)heated lysozyme solutions (10 g L^−1^) was adjusted with 0.5 M HCl to pH 3.0, and subsequently with 0.5 M NaOH to pH 7.0, 7.5, 8.0, 8.5, 9.0, 9.5, 10.0, 10.5 and 11.0.

Turbidity: The turbidity was determined based on the absorbance at 595 nm.

Solubility: The solubility was determined based on absorbance at 280 nm and on nitrogen content by Dumas. Prior to analysis, the solutions were centrifuged (16100× *g*, 10 min) and, for the absorbance measurement, diluted 20 times with 10 mM sodium phosphate buffer pH 5.7. The protein concentrations were calculated using a molar extinction coefficient of 37,970 M^−1^ cm^−1^ [53] and a nitrogen conversion factor of 5.29 [47].

### 3.11. Adsorption Kinetics

The (un-)heated lysozyme solutions were diluted to a final concentration of 0.1 g L^−1^. The surface tension as a function of time was measured using an automated drop tensiometer (ADT, Teclis IT Concept, Longessaigne, France). The system was temperature-controlled at 20 °C and the bubble volume was kept constant at 7 µL for 3600 s. The change in surface tension compared with that of the pure interface (i.e., the air–water interface) was expressed as the surface pressure (Equation (4)) [54].

(4) $$\Pi \left(t\right)=\gamma_{0}-\gamma \left(t\right)$$

where γ_0_ is the measured interfacial tension of the buffer [72.0 mN m^−1^]. The lag time was defined as the time required to reach a surface pressure of 1.0 mN m^−1^.

The surface elastic modulus (E_d_) was measured by inducing sinusoidal changes in the interfacial area with an amplitude of 5 % of the bubble area and a frequency of 0.1 Hz. The modulus was calculated from the measured changes in surface tension and surface area averaged over a sequence of five sinuses using Equation (5) [55]. Every 100 s, a sequence of five sinuses was performed. All measurements were performed in duplicate.

(5) $$\left|E_{d}\right|=\frac{\Delta \gamma }{\left(\frac{A}{\Delta A}\right)}$$

where Δγ is the amplitude of the surface tension oscillation [mN m^−1^] and A and ΔA are the bubble area and amplitude in bubble area [mm^2^], respectively.

### 3.12. Foam Formation and Stabilization

The foam of the (un-)heated lysozyme solutions at concentrations of 0.05–10 g L^−1^ was made by sparging N_2_ into the protein solutions in an automated foaming device (Foamscan, Teclis IT-Concept, Longessaigne, France). Nitrogen was sparged into 60 mL protein solution at a flow rate of 400 cm^3^ min^−1^ to a final foam volume of 400 cm^3^. The temperature in the foam tube (diameter 60 mm) was controlled at 25 ± 1 °C. The foam cylinder was equipped with a metal frit (average pore size 28 ± 2 µm, spaced in a hexagonal lattice with 100 µm distance between the centres of the pores).

The relative foam volume was determined by comparing the foam volume of the (un-)heated lysozyme solutions after 60 s of sparging with the maximum obtainable foam volume after 60 s. The maximum obtainable foam volume was determined to be 339 cm^3^ using a 10 % (*w*/*v*) solution of sodium dodecyl sulphate (SDS). The average Sauter mean radius (r_3,2_) of the bubbles was determined by image analysis of foam images taken 1 s after the end of sparging at a height of 8 cm above the protein solution, which corresponds roughly to the middle of the foam column. The minimum bubble radius (r_3,2, min_) depends on the system parameters, such as flow rate, pore size, and liquid volume, and was, for a 10 % (*w/v*) sodium dodecyl sulphate solution under the current settings, determined to be 0.06 mm. Based on the determined foam volume and average bubble radius, the amount of created surface area (A_tot_ in m^2^) was calculated (Equation (6)) [3]. For the calculation, it was assumed that the bubbles are spherical and homogeneous throughout the foam and that the bubbles completely fill the volume of the foam.

(6) $$\begin{array}{cc} A_{\mathrm{tot}} & =4{\pi R}_{\mathrm{bubble}}^{2}{\#}_{\mathrm{bubbles}}=4{\pi R}_{\mathrm{bubble}}^{2}\frac{V_{\mathrm{foam}}}{V_{\mathrm{bubble}}}\\ & \;\;\;\;=4{\pi R}_{\mathrm{bubble}}^{2}\frac{V_{\mathrm{foam}}}{\frac{4}{3}{\pi R}_{\mathrm{bubble}}^{3}}=\frac{3V_{\mathrm{foam}}}{R_{\mathrm{bubble}}} \end{array}$$

where R_bubble_ is the bubble radius [m], #_bubbles_ is the number of bubbles [-], and V_foam_ and V_bubble_ are the total volume of the foam and the volume of a bubble [m^3^], respectively.

The foam volume over time (until t = 10,000 s) was monitored by a camera. The foam stability is expressed as of the foam half-life time (t_1/2_), i.e., the time needed for 50% of the foam volume to collapse. The collapse of all foams was gradual and symmetrical, i.e., the top of the foam stayed more or less horizontal during collapse. Reported values of foam ability, r_3,2_ and t_1/2_, are averages of at least two individual foam experiments.

## 4. Conclusions

A protein’s exposed hydrophobicity strongly affects foam formation through its effect on the adsorption barrier and the consequent change of the adsorption rate constant. To be more specific, with increasing exposed hydrophobicity, the adsorption rate constant increases, thereby enhancing foam formation (i.e., increased foam volume and/or smaller bubbles). At higher exposed hydrophobicity, the adsorption rate constant and foam formation become independent of hydrophobicity. In contrast to foam ability, foam stability (i.e., foam collapse) was not affected by hydrophobicity (under identical conditions). At a given concentration in the protein-poor regime, an increased exposed hydrophobicity will indirectly result in a higher foam stability, through its effect on the foam ability. The observed relation between exposed hydrophobicity and foam ability underlines the similarity between the behaviour of protein-stabilized foams and emulsions, and thereby shows the potential of using this model to describe foam ability in the future.

## Figures and Tables

**Table 1:** Relative exposed hydrophobicity (Q_H_), lag time, and critical concentration for foam ability (C_cr, FA_) and bubble size (C_cr_, r32) for (un-)heated lysozyme.

	Relative Q_H_ [-]	Lag Time [s]	C_cr, FA_ [g L^−1^]	C_cr, r32_ [g L^−1^]
Lys0	0.20	2135	>10	>10
Lys15	0.37	1685	5	>10
Lys30	0.67	960	2.5	10
Lys60	2.28	580	1	5
Lys90	6.33	80	1	5
Lys120	9.90	60	0.5	2.5

## Data Availability

Not applicable.

## Supplementary Materials

The following supporting information can be downloaded at: https://www.mdpi.com/article/10.3390/molecules27072358/s1, Figure S1. Size-exclusion chromatogram (A) and mass spectra (B) of the (un-)heated lysozyme variants; Figure S2. Thin liquid film rupture times (after 5 min aging) as a function of concentration for (un-)heated lysozyme; 0 (◇), 30 (△), 60 (×), 90 (🞶) and 120 min (○; A) and thin liquid film rupture times (after 20 min aging) for (un-)heated lysozyme at 0.25 g L^−1^ before () and after (◆) exchange of the interior protein solution with buffer (B) (10 mM sodium phosphate buffer pH 5.7, 25 °C). The lines in panel B are guides to the eye. It should be noted that these lysozyme variants are heated at 75 °C rather than 77 °C.

## Sample Availability

Samples of the compounds are available from the authors.
