# Supplementary material for: Hydrophobicity Enhances the Formation of Protein-Stabilized Foams
Source: Molecules. 2022 Apr 6;27(7):2358. doi: 10.3390/molecules27072358 (PMC9000900; doi:10.3390/molecules27072358)
Supplement: Supplementary file 1 [file molecules-27-02358-s001.zip › molecules-1638841-supplementary.pdf]

Supplementary information

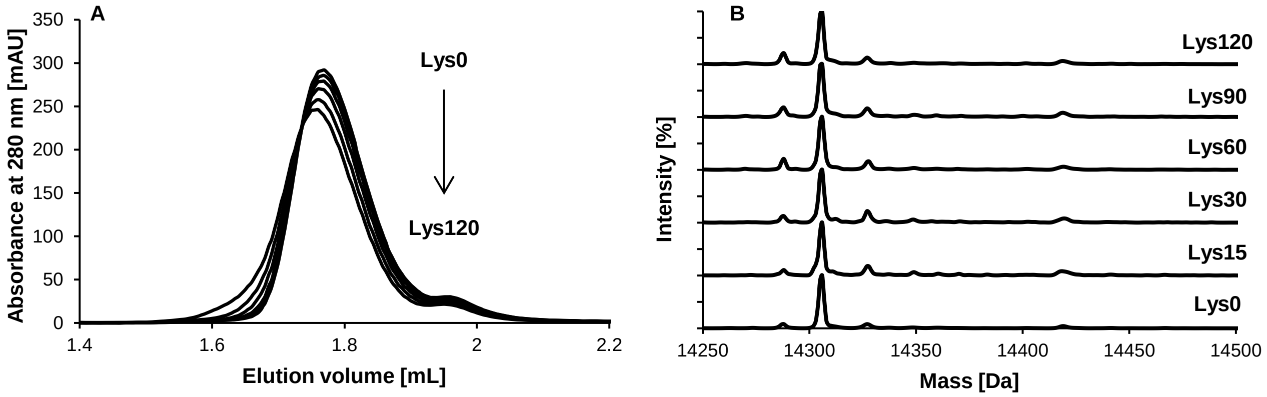

Figure S1. Size-exclusion chromatogram (A) and mass spectra (B) of the (un-)heated lysozyme variants.

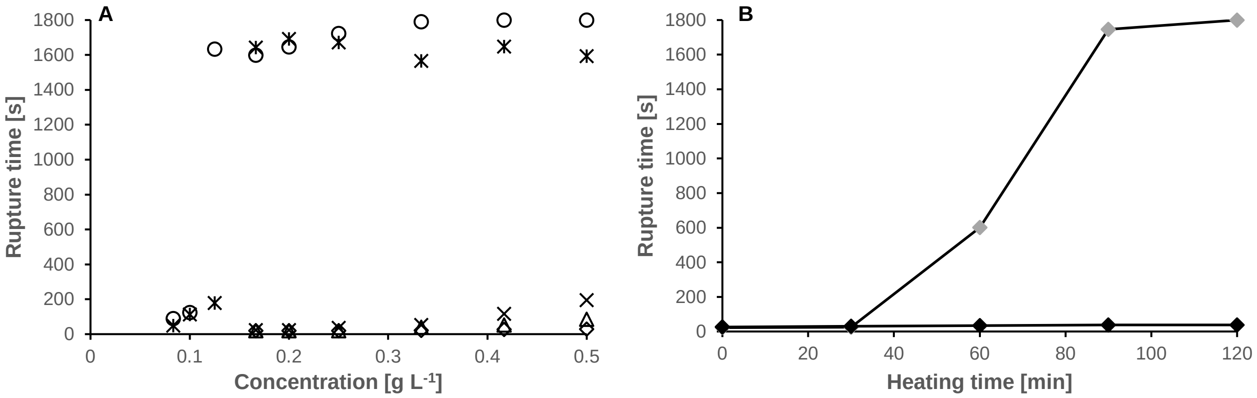

Figure S2. Thin liquid film rupture times (after 5 minutes aging) as a function of concentration for (un-)heated lysozyme; 0 (◇), 30 (△), 60 (×), 90 (∗) and 120 minutes (○; A) and thin liquid film rupture times (after 20 minutes aging) for (un-)heated lysozyme at 0.25 g L^{-1} before (◆) and after (◆) exchange of the interior protein solution with buffer (B) (10 mM sodium phosphate buffer pH 5.7, 25 °C). The lines in panel B are guides to the eye. It should be noted that these lysozyme variants are heated at 75 °C rather than 77 °C.
